# Planning resource allocation for husbandry management by portfolio optimization

**DOI:** 10.1016/j.heliyon.2022.e10841

**Published:** 2022-10-05

**Authors:** Todor Stoilov, Krasimira Stoilova, Stanislav Dimitrov

**Affiliations:** Institute of Information and Communication Technologies, Bulgarian Academy of Sciences, Bulgaria

**Keywords:** Portfolio optimization, Risk management, Decision making, Resource allocation

## Abstract

The husbandry management is assessed in general by comparison of current and past economical results, which are used as a universal business metric. Sustainable management in general targets minimization of risk and maximization of the return for managing business activities. The minimization of the economic risk allows for decreasing the potential losses for the husbandry management and they are leading criteria for planning future resource allocations. The new point added in this research concerns simultaneously inclusion in the portfolio problem the risk formalization both as a standard deviation of return and the probability for losses as value-at-risk. Several portfolio problems are defined, considering the probability of losses as a goal function or constraint in the portfolio problems. The inclusion of these two formalizations allows the portfolio risk to decrease additionally in comparison with the classical portfolio problems, where the risk is quantified as a standard deviation of the portfolio return. The peculiarities of these problems and the corresponding optimal solutions are analyzed, which allows quantifying the resource allocation per different business activities. Numerical experiments are made with real data on animal husbandry, available from the Bulgarian National Statistics and the results are illustrated in a graphical way. The empirical comparison with these data gives benefits in decreasing the risk when both risk formalizations are applied in the portfolio problem.

## 1 Introduction

The portfolio theory is a powerful tool for decision-making in investment and resource allocation. Its domain of application is extended not only in financing but also to management in different areas of applications. The paper addresses the optimization of resource allocation in husbandry management. The resource allocation for the husbandry management and the decision-making has internal complexity because they have to respect a set of criteria and requirements. In general, the planning of resource allocation needs quantified solutions, which are the reasons, the decision-making process to be formalized as an optimization problem. This research does not make an extended overview of the formal approaches, applied for decision making, but it illustrates the application of the portfolio theory for optimal planning resources in husbandry management. The recommendations for resource allocation are made by definition and solution of a portfolio optimization problem, which targets maximization of the return and minimization of the management risk (Khan et al., 2020). The portfolio theory was chosen for this research because the economic value of husbandry management can be used as a universal metric for optimal decision-making in investments and resource allocation (Dobrowolski et al., 2022).

The application of the portfolio approach targets simultaneously maximization of the portfolio return and minimization of the portfolio risk. The risk is quantified in a way as a standard deviation of the volatility of the return around its mean (Khan et al., 2020). The elaborations for quantification of the risk category for considering more parameters of the portfolio led to the definition of risk as a probabilistic value for potential losses (Aven, 2016, Liu, 2020). The management of animal husbandry targets the increase the profit from the exploitation and outcomes of its production. The resource allocation is strongly based on these management results. But risk management is an important part of increasing the profit. Decreasing the risk is a prerequisite for sustainable financial management and investment decision-making (Pyka and Nocoń, 2021). The husbandry management has to follow simultaneous minimization of the risk and maximization of the return from the husbandry production. Husbandry production in general contains several components, which give different returns. The total return from the exploitation of the husbandry is the main source for additional improvements in husbandry production. Thus, the increase in the return is the main goal for the management and to allocate additional resources for husbandry production. But the operation and management face different events, which originate risk for successful and safe production and exploitation. The risk events can deteriorate as a result of husbandry exploitation. Risks take place both for income and for the disbursements of the livestock production. These events affect the management decisions and their consequence cannot be predicted accurately. The appearance of random events and their influences on husbandry production originates the existence of risk. Respectively, this influence of risk on production parameters has to be assessed and manage appropriately in the husbandry operations. The sources of risks can originate from different conditions. The weather conditions can change the normal policy of feeding livestock production. The market price fluctuations can change prices for livestock production, crop sales, and equipment support. The changes in government policies for subsiding influence the current financial management of the husbandry. Risks exist for the support of the well-being of animals, the costs of labor, and other related activities, which take place in animal husbandry management. All these events and in combination can randomly change the husbandry returns from the production components. This is the reason the husbandry management optimizes the allocation of resources supporting the production of its outcomes, targeting maximization of the returns and considering decrease and/or minimization of risks, originated from different events.

An approach for decreasing the risk is the policy of diversification in production (Lee et al., 2020). But the approach applied in this research is based on the formal background of the portfolio theory (Kolm et al., 2014). The portfolio theory is mainly applied to the financial domain. But there are many outside activities, which are formalized in the background of this theory. This is due to the quantified approach, which the portfolio theory applies for the general case of decision-making in investment and resource allocation. Examples of such applications one can find for the cases: management sciences (Levy and Lim, 1994); product portfolio management (Doorasamy, 2015); marketing (Brown, 2010); environmental sciences (Matthies et al., 2019); climate change (Crowe and Parker, 2008); environmental policy (Antal, 2008); energy policy (deLlano-Paz et al., 2017); water management (Marinoni et al., 2008); water planning (Beuhler, 2006); fish population (DuFour et al., 2015); real estate portfolio management (Souza, 2014); agricultural sciences (Barkley and Hanawa, 2008); portfolio for biodiversity (Figge, 2004); agronomy (Radulescu et al., 2014); health care (Fagefors and Lantz, 2021); project portfolio management (El Hannach et al., 2019). The portfolio approach is applied also for inventory management and the risk is formalized in probabilistic forms (Zhi et al., 2021). The portfolio formalization is used for the evaluation of the resource allocation per set of projects (Özpeynirci et al., 2022).

The portfolio theory provides in general quantitative solutions for the domains of decision-making in investments and resource allocation. This is a prerequisite for sustainable management nevertheless of the application areas. The reason for the choice of the portfolio theory for this research is motivated by sustainable husbandry management, based on optimal decision-making, based on the universal metric as investments and resource allocation (Dobrowolski et al., 2022).

The goal of this research is to apply the portfolio formalization for recommended resource allocation per different husbandry productions. The portfolio problems consider in an explicit way the risk in the management policy. An extensive overview of the risk existence and its form for quantification one can find in (Meyer, 2015). The portfolio theory applies the risk in classical statistical forms as a standard deviation of the portfolio return. Another additional form of risk is the value of the potential loss, which is quantified as the parameter value-at-risk. The solutions of the portfolio optimization target simultaneously maximization of the returns and minimization of the different formalization of risk. This research applies modifications of the portfolio problems by simultaneous usage of two formalizations of the portfolio risk: classical standard deviation and the parameter value-at-risk. The inclusion of these two formalizations allows the portfolio risk to decrease additionally in comparison with the classical portfolio problems, where the risk is quantified as a standard deviation of the portfolio return. Because the value-at-risk parameter is described as probabilistic inequality, its inclusion in the portfolio problem is performed by approximation of the probabilistic inequality to an algebraic one. Then several portfolio problems are defined, which takes into consideration the value at risk formalization as a goal function or constraint in the portfolio problem. The portfolio problems optimize both the husbandry returns and simultaneously consider appropriate levels of risk. These models give optimal solutions for the allocation of resources per different productions, which will increase the total husbandry outcome and simultaneously provide minimization of the management risk.

The paper is organized as follows. In section 2 we derive the formal definitions of the risk in statistical forms of the standard deviation of return and a probabilistic description of the potential loss. In section 3 the portfolio models are defined with additive and nonlinear goal functions for simultaneous maximization of the return and minimization of the risk considering its two forms of quantification. In section 4 we approximate the probabilistic inequality for the definition of the risk in algebraic inequality form. In section 5 a set of optimization problems is defined where the risk is evaluated in both its form and is applied as goal functions or constraints in portfolio problems. The comparisons are discussed in section 6 with graphical interpretations of the results of the empirical study based on real statistical data. In section 7 an assessment of the potential of the modified portfolio problems and the ways for additional modifications are given.

## 2 Overview of formal definitions of the risk in husbandry management

The sources of risk in livestock enterprises are described and analyzed in (Chand et al., 2018). In (Kahan, 2008) extensive analysis of the types of risk and their assessment is given. Particularly, in animal husbandry, the risks in breeding and rearing animals are an important part of the livestock production in husbandry management (OIE, 2021). Researches and recommendations about the management of risk in farming are in focus for the husbandry exploitation (Harwood et al., 1999; Deloitte, 2017). It is stated that the management of farms becomes more commercial, which is a requirement to assess this management from point of view of commercial criteria (Rehman et al., 2017). The management of the husbandry targets sustainable development and predictive results in their exploitation (Win et al., 2019; Scialabba et al., 2014). Sustainable development is targeted also with the application of information and communication technologies (El Bilali and Allahyari, 2018). These technologies allow the management of husbandry to be performed with quantification and application of logical and formal models, which are prerequisites for achieving optimal results in the husbandry exploitation for maximization of production and returns and minimization of exploitation costs. Quantification approaches are recommended and applied in livestock management (Win et al., 2019) and in conservation tasks in husbandry management (Garcia-Diaz et al., 2019). The husbandry management targets prospective development in stock production and economical outcomes (Obućinski et al., 2019, Ishchenko et al., 2020). Husbandry management is assessed in an integral form by economic criteria (Flaten et al., 2005). The economic state of husbandry and its development can be quantitatively assessed by various criteria. These criteria quantify the variables and parameters of the husbandry exploitation. The key issue is to provide quantification of the components, which results from the exploitation tasks of the husbandry management. Such types of criteria, variables, and parameters in animal husbandry management are extensively discussed in (Woodend, 2010). This research takes into consideration only economic criteria for the management, which present in integral form the quality of the husbandry management. The risk-averse management of enterprises is an important policy, which must be followed in financial decision-making (Gilbert and Meiklejohn, 2019). Following a sequence of decomposition of terms, the financial components of outcomes of the animal husbandry production from rearing and breeding, a set of tasks are summarized in Table 1.

**Table 1:** Examples of rearing and breeding financial outcomes in an animal husbandry.

Incomes	Disbursements	Debt obligations
*Livestock sales*	*Fodder*	*Building loans*
*Crops sales*	*Drugs*	*Farmland mortgage*
*Government payments*	*Machine, Equipments*	*Equipment loan*
*Others*	*Labor*	
	*Property taxes*	
	*Insurance*	
	*Family living*	

In general, the husbandry returns result from the incomes and disbursements, which occur for the husbandry management (Gönsch, 2017). The incomes concern direct livestock sales, secondary production sales like milk, meat, and others, and miscellaneous cash flows from rents, interests, hiring out machinery, labor, and others. The disbursements include general livestock purchases, inventory management (crops, welfares, others), and miscellaneous (insurance, payments, labor, equipment, family living). The difference between the incomes and disbursements gives a quantitative assessment of the husbandry return and it is a value of the performance of animal farming. The role of quantification in the management of incomes and disturbance in husbandry is an important task, which has to be used in farm decision-making (Dimitrov et al., 2021). But the manner of formal, quantitative assessment depends on the nature of incomes and disturbances: deterministic or probabilistic. The majority of the described components in the hierarchy of income and disbursement indicators have a stochastic nature. This results in the stochastic nature of the final value of the husbandry return, which is the main criterion for the level of performance of its management. The random character of the return requires its assessment and identification to be performed not only by its value but also by the risk, which is related to the stochastic variables (Flaten et al., 2005; Zopounidis et al., 2018).

The analytical formalization of management decisions in animal husbandry can be performed by usage of relations from the modern portfolio theory. The last derives models, which consider simultaneously both the requirements for increase of the portfolio return and decreasing the portfolio risk. For this research, the risk is considered in the framework of the portfolio theory, which provides quantitative responses for decision-making. The formalization and the quantification of the risk indicators are successfully applied in the portfolio theory (Sharpe, 1999; Janabi, 2019; Malz, 2011; Du Plessis, Van Rensburg, 2020). The risk has a stochastic nature and its management has to consider this random behavior (Šimovi'c and Tafro, 2021, Guo et al., 2021). The statistical characteristics of a stochastic variable *R*_*i*_(*t*) and according to the “Empirical rules” the percentage of the stochastic values of *Ri* (*t*) that lie under the probability density function for a normal distribution process according satisfy the relations (URL1).

*Pr* (*E*_*i*_ − σ_i_≤ *Ri* (*t*) ≤ E_i_ + σ_i_) ≈ 68,27%

*Pr* (*E*_*i*_ − 2σ_i_*≤ Ri* (*t*) *≤ E*_*i*_*+* 2σ_i_) *≈* 95,45%

**Figure 1:**
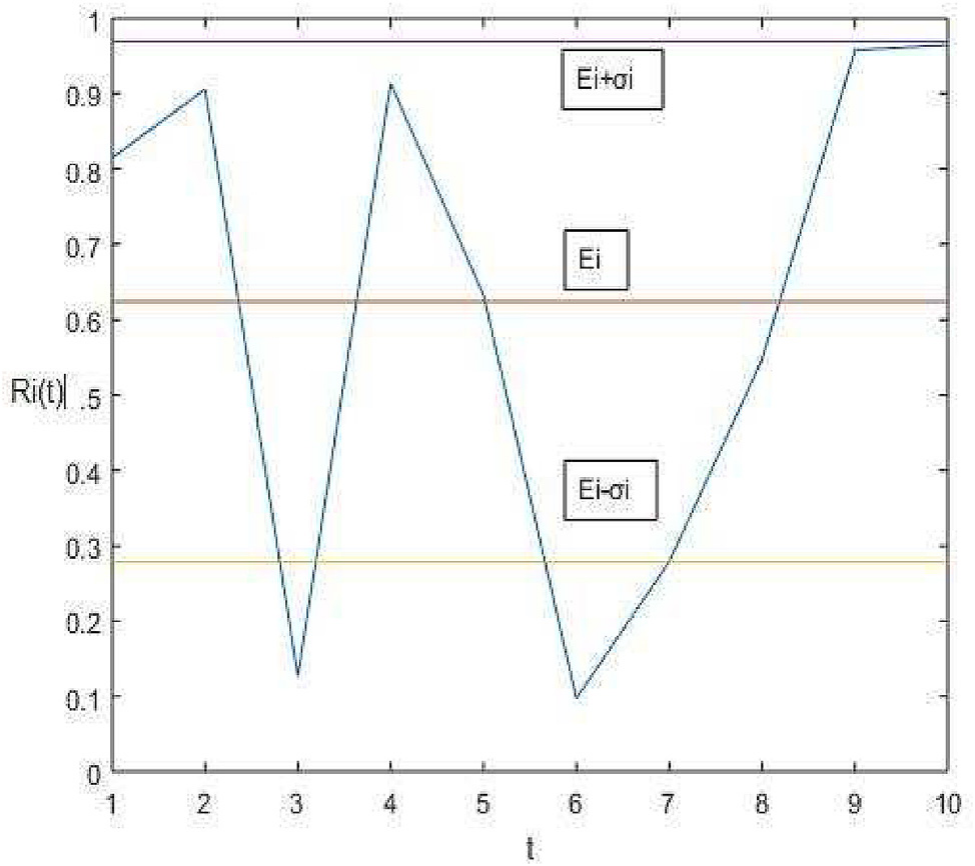
Relations between the real value of return *R*_*i*_ (*t*) and its lower and upper bounds [*E*_*i*_ − $\sigma_{i}$*, E*_*i*_*+*$\sigma_{i}$].

*Pr* (*E*_*i*_ − 3σ_i_*≤ Ri* (*t*) *≤ E*_*i*_*+* 3σ_i_) ≈ 99,73%*,*

where the notation *Pr()* means probability*,* the value *E*_*i*_ is the mean level of the stochastic variable for a period in question, and $\sigma_{i}$ is the standard deviation, which quantifies the risk, Figure 1. The practical occasions apply the first inequality, which consider the predominantly changes of *Ri*(*t*) in the diapason [*E*_*i*_ − $\sigma_{i}$*, E*_*i*_ *+* $\sigma_{i}$].

If the risk $\sigma_{i}$ has a small value, hence the range around the mean value *E*_*i*_ will be small and the real value of the stochastic variable *R*_*i*_ *(t)* will be close to the mean *E*_*i*_. This will benefit the estimation of the real value of *Ri (t*) and make easy its forecast for decision-making. In the opposite case, if the risk $\sigma_{i}$ has a big value, this makes it difficult to forecast the real value of the stochastic variable, because it can change in a wide area around the mean *E*_*i*_. Hence, the real value of *R*_*i*_(*t*) can be considered as an intermediate value between the upper and lower bounds of the mean *E*_*i*_*,* [*E*_*i*_ − $\sigma_{i}$*, E*_*i*_ *+* $\sigma_{i}$]. This case is not favorable for big $\sigma_{i}$ for the husbandry management and this defines that there is considerable risk for the real return from the farm management. The graphical interpretation of the risk, as an area around the mean value of the parameter in question, is given in Figure 1.

After evaluating the portfolio solutions, the real value of the portfolio return *R*_*i*_ *(t)* will belong to the area [*E*_*i*_ − $\sigma_{i}$*, E*_*i*_ *+* $\sigma_{i}$]. But this value can be considerably different from the estimated mean value *E*_*i*_ in the case of big risk $\;\sigma_{i}$.

The formal analytical relations for the evaluation of the mean value and risk of a stochastic variable have linear and quadratic forms. For a set of *N* random variables *R*_*i*_ *(t)*, their values are recorded in a discrete set of sequences of *n* values in time,

(1) $$\begin{array}{l} R_{1}=\left[R_{1}^{\left(1\right)},R_{1}^{\left(2\right)},\ldots ,R_{1}^{\left(n\right)}\right]\\ \ldots \\ R_{N}=\left[R_{N}^{\left(1\right)},R_{N}^{\left(2\right)},\ldots ,R_{N}^{\left(n\right)}\right]\\ R=\left[R_{1},\;\ldots ,\;R_{N}\right] \end{array}$$

These records Eq. (1) allow the evaluation of each mean value $E_{i}\;$ for the *N* variables and the corresponding volatilities $\;\sigma_{i}^{2}$ for the defined time interval 1 ÷ *n*

(2) $$\begin{array}{l} E_{i}=\frac{1}{n}\overset{n}{\underset{k=1}{\sum }}\;R_{i}^{\left(k\right)},\;\\ \sigma_{i}^{2}=\frac{1}{n}\overset{n}{\underset{k=1}{\sum }}\;{\left(E_{i}-R_{i}^{\left(k\right)}\right)}^{2},\;i=1,\ldots N\;. \end{array}$$

Thus, the risk of the stochastic variable *R*_*i*_ *(t)* is numerically estimated as a standard deviation $\;\sigma_{i}$, Eq. (2).

Another form of quantification of the risk is given by probability inequality, which is applied with the parameter Value-at-Risk (VaR) (Dowd, 2005). The VaR parameter quantifies the likely loss for the returns from portfolio investments in assets. The VaR is an index for the risk of a stochastic variable. Currently, this index is accepted as a new risk indictor, which assessment and management provide many positive effects for the decision-making (Chen, 2018; Menéndez and Hassani, 2021). VaR gives a quantitative level of the risk in terms of maximum likely loss (Janabi, 2019). It exists an attempt to apply a VaR form of risk in inventory modeling (Luciano et al., 2003; Zhi et al., 2021). The quantification of risk in VaR formal definition is applied in supplied chain networks (Khorshidi and Ghezavati, 2019). The assessment and usage of VaR formalization are accepted as a prospective manner for risk management (Luciano et al., 2003; Josaphatand and Syuhada, 2021).

**Figure 2:**
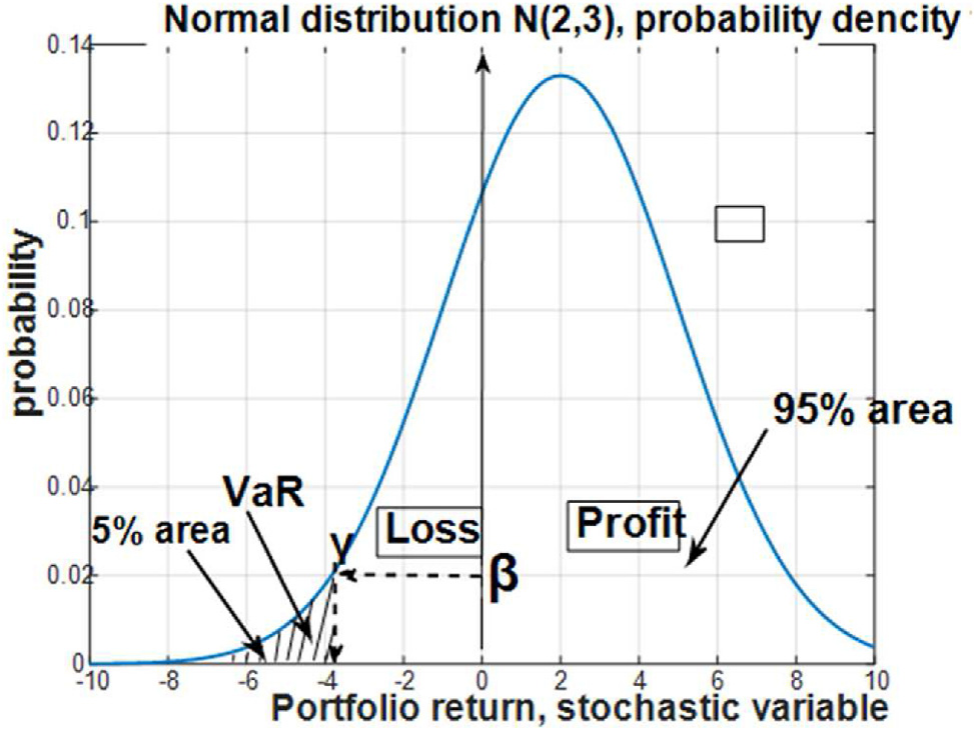
The density probability function of the stochastic variable of animal husbandry return/loss.

The formal presentation of the value of VaR is given in Figure 2. It is presented by the density probability function of a husbandry index, representing the return and loss variable of the husbandry management.

The positive value of the portfolio return is the profit from the husbandry management, while the negative value is the loss (Rutkauskas and Stasytytė, 2020). The quantity of the portfolio loss is γ, and the probability of having γ losses is ***β***. From Figure 2 it is presented that the value of losses VaR = γ can be expressed by the density probability function *f* (**X**) of a random variable **X** and the required level of probability ***β*** (Kuester et al., 2006)

$$VaR=\overset{\beta }{\underset{0}{\int }}f\left(y\right)dy=\;\gamma$$

**Figure 3:**
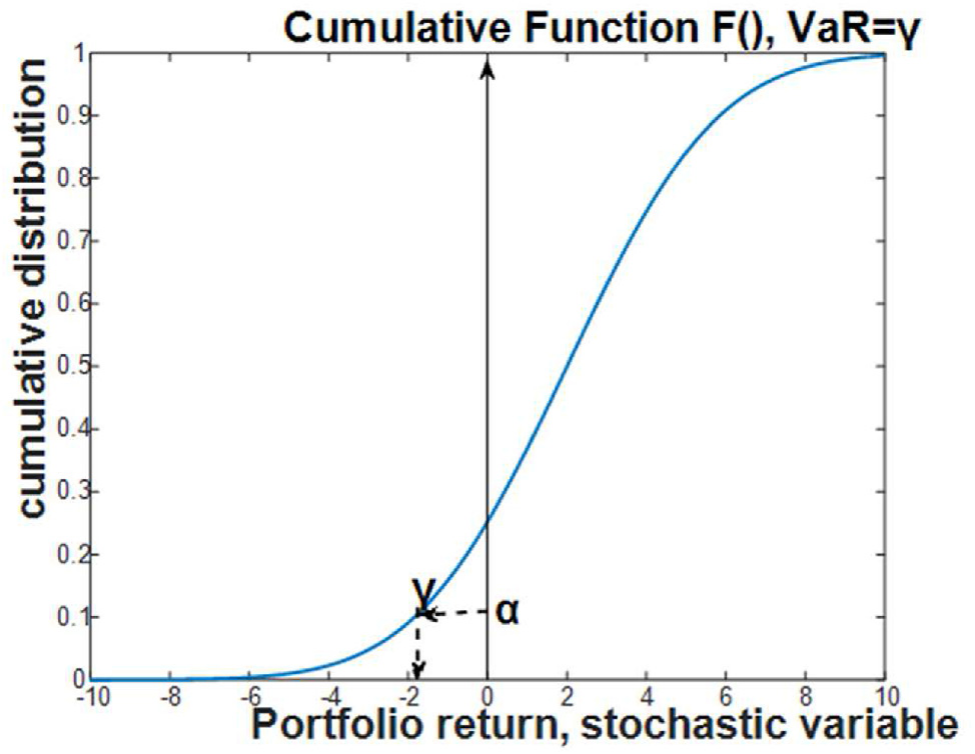
Graphical interpretation of VaR with cumulative density function.

The same expression can be rewritten with the cumulative probability function *F*(x) of the stochastic variable X and the value of VaR = γ, which is illustrated in Figure 3. The index VaR took different modifications and forms, which are titled “liquidity-adjusted value-at-risk” (LVaR) and “conditional VaR” (CVaR) (Al Janabi, 2019; Ranković et al., 2016).

The formal analytical relation for the value of VaR is given by the probabilistic inequality following Figure 2 as

(3) VaR (Y) = min {γ: P (Y ≥ γ) ≤ 1 − *****β*****}.

The interpretation of this relation says that the husbandry loss in return Y will be the minimal number γ, which corresponds to the required level of probability ***β***. Hence, the value of VaR is defined by both parameters: the level of the losses γ and the probability ***β***, required for occurring this loss. Relation Eq. (3) can be expressed also in the additional form of probabilistic inequality, considering that the probability takes values from 0 to 1,

(4) P (Y ≤ γ) ≥ ***β***

These formalizations are applied by the portfolio theory. This gives ground for the application of this theory in decision making and husbandry management, targeting maximization of the return and minimization of risk. The application of elements of the portfolio theory can be found in (Šimovi'c and Tafro, 2021), where the portfolio components are applied to inventory policy. Particularly, the risk is formalized with probabilistic relations in the form Eq. (4) of VaR. Such a probabilistic form of risk is used by (Xin-shu and Wei, 2007) for decision-making in inventory control in farm management. In (Khorshidi and Ghezavati, 2019) the VaR parameter is applied for quantification of risk in husbandry product distributions.

The VaR parameter has internal limitations (URL2). The great number of stochastic variables makes its evaluation difficult. It is used different approaches for the evaluation of VaR, which can give various values for the same portfolio. The time period, for which VaR is evaluated has to conform with the historical data about the returns.

In this research, the portfolio problems are modified by the application of VaR relations as goal function or constraint for the portfolio problem. In this case, the losses γ and the probability ***β*** are given as parameters for the portfolio problem. The solutions of the problem define the relative amounts of the resource allocations. These modified portfolio problems are derived in the next section.

## 3 Portfolio optimization problems for risk management

The approach, which is followed for the definition of optimization problems for resource allocation and risk management originated from the portfolio theory (Gilbert and Meiklejohn, 2019). The problem, which is used from the portfolio theory, provides minimization of the portfolio risk and maximization of the portfolio return. This optimization problem has an analytical form as

(5) $$\min_{w}\left\{\left(1-\lambda \right)w^{T}\Sigma w-\lambda E^{T}w\right\}$$

$$w_{1}+w_{2}+\ldots +w_{N}=1\;\text{or}\;w^{T}\left|1\right|=1,\;w_{i}\geq 0,\;i=1,\ldots ,N$$

where,

*E*_*i*_–the mean return of the of the category *i =* 1,*…,N*, E^T^ = (*E*_*1*_*, …, E*_*N*_),

Σ–the covariance matrix between the returns of all categories,**w**^**T**^ =(*w*_*1*_*, …, w*_*N*_), *w*_*i*_ –the relative part of resources (weights), which are recommended for allocation to the different categories of returns, *i =* 1,*…, N*. This vector is the solution to the optimization problem Eq. (5), which recommends how many resources are allocated per production category *i* by means to achieve a maximal return from the entire husbandry management and keep a low level of risk.

$E^{T}w$—this value defines the total return from the husbandry management, which is a sum of all local return components *E*_*i*_*w*_*i*_, obtained with *w*_*i*_ allocated management resources.
$w^{T}\Sigma w$—this value gives quantification of the total risk for husbandry management. It is a quadratic relation between the solutions **w** and the covariance matrix. The last is a quadratic, symmetric matrix *NxN.* The elements on the diagonal are the volatilities of the different local returns, which risk is quantified with the value $\sigma_{i}^{2}$. The non-diagonal elements of Σ are values of the correlations between the different local returns, which influence also the total risk of the husbandry management. The elements *cov*_*ij*_ between the couples of individual returns i,j∈1,…,N are evaluated according to Eq. (6) with the available data from Eq. (1)

(6) $$cov_{ij}=\frac{1}{n}\sum_{k=1}^{n}\left(R_{i}^{\left(k\right)}-E_{i}\right)\left(R_{j}^{\left(k\right)}-E_{j}\right),\;\forall i,j\in 1,\ldots ,N$$

$$cov_{ii}=\sigma_{i}^{2},\;cov_{ij}=cov_{ji},\;\forall i,j\in 1,\ldots ,N.$$

λ is a coefficient, which defines the ability of the decision-maker to undertake risk. This coefficient takes values from the set [0, 1]. If λ=1, the decision-makers target only maximization of the return, while for λ=0 he minimizes only the risk of the husbandry management. The values of λ, inside the feasible set, correspond to the subjective abilities of the decision-maker to give priority to the risk or to the return.

Solving Eq. (5) for different values of λ, different solutions **w** (λ) are evaluated and the corresponding values of the total management return $E^{\text{T}}w\left(\lambda \right)$ and risks $w^{\text{T}}\left(\lambda \right)\Sigma w\left(\lambda \right)$ are presented as a sequence of points in the space *Risk(Return)*. This set of points is named “efficient frontier” and it is recommended for the decision-makers to choose one point from this curve, which will define its problem solution.

In this research, we are going to choose this point as a solution to a problem Eq. (5), which gives the minimum value of the relation *Risk* toward the *Return*

(7) $$\begin{array}{l} min\\ w \end{array}\frac{w^{T}\left(\lambda \right)\Sigma w\left(\lambda \right)}{E^{T}w\left(\lambda \;\right)}$$

Relation Eq. (7) is used as a goal function for a nonlinear portfolio problem

(8) $$\begin{array}{l} \begin{array}{l} min\\ w \end{array}\frac{w^{\text{T}}\Sigma w}{E^{\text{T}}w}\\ w^{T}\left|1\right|=1,\;w^{T}\geq 0 \end{array}$$

These, two optimization problems, Eq. (5) and Eq. (8) perform the minimization of the risk of husbandry management and simultaneously maximize the return. The risk is defined as a standard deviation of the return and it is used as a goal function for the portfolio problem. Problems Eq. (5) and Eq. (8) do not contain formal relations for considering losses by the VaR parameter. The solutions of these problems give the allocation of the relative values of the management resources for different activities in husbandry management. Both problems perform maximization of return and minimization of risk for the management. The difference between Eq. (5) and Eq. (8) comes from the analytic description of the portfolio goal function. Problem Eq. (5) insists additional definition of the parameter λ, which defines the subjective ability for undertaking risk. The solutions to these two problems are considered as benchmarks for comparisons with the cases when the risk is formalized in the form of VaR.

The parameter VaR for the risk was given with the probabilistic inequality Eq. (4). For case Eq. (4) to be included in a portfolio problem this research makes an analytical approximation of Eq. (4) in a form of algebraic inequality. The derived relation is added to problems Eq. (5) and Eq. (7), which give new modifications of the portfolio problem for the optimal allocation of the management resources.

## 4 Approximation of the VaR relation in an algebraic relation

We are going to start with relation Eq. (4) for the probabilistic definition of the value of VaR = γ, where γ is the level of losses. By multiplication with (−1), both sides of the inequality in the probabilistic operator P (.) from Eq. (4) take the forms

(9) P (−Y ≥ −γ) ≥ *****β*****,

where β is the level of probability for occurring losses, which are higher than the predefined volume of γ. The value of β is subjectively chosen by the decision-maker in accordance with the time horizon under which the resource allocation is planned.

The stochastic variable Y concern losses, but for the case of management, we evaluate the return $R^{\text{T}}w$ of the management, which is also a stochastic value. Hence, the losses are quantified by a negative value of return, $R^{\text{T}}w=-\text{Y}$, and the probabilistic relation Eq. (9) becomes

(10) $$\;\text{P}\left(R^{\text{T}}w\geq -\gamma \right)\geq \beta$$

Relation Eq. (10) cannot be directly used in a portfolio problem, which is the reason to derive its analytical approximation. Such approximation of Eq. (10) is performed by a sequence of rules, which normalizes both sides of the probabilistic inequality. Thus, the stochastic process $R^{\text{T}}w$ is modified with zero mean and standard deviation equal to 1. For the normalization, the mean value $E^{\text{T}}w$ is subtracted from the left and right sides of the probabilistic operator P (.) and they are divided by the standard deviation $\sqrt{w^{\text{T}}\Sigma w}$, and Eq. (10) is rewritten like

(11) $$\;\text{P}\left(R^{\text{T}}w\geq -\gamma \right)=\text{P}\left(\frac{R^{\text{T}}w-E^{\text{T}}w}{\sqrt{w^{\text{T}}\sum w}}\right)\geq \frac{-\gamma -E^{\text{T}}w}{\sqrt{w^{\text{T}}\sum w}}\beta .$$

Implicitly we concern with normal distribution for the stochastic process $R^{\text{T}}w$**.** Now we apply the relations between the density function P (.) and cumulative one F (.) as

F (γ) = P (**R**^T^**w** ≥ −γ) ≥ *****β***** or 1 – F (γ) = P (**R**^T^**w** ≥ −γ) ≤ *****β*****

and relation Eq. (11) becomes

$$\text{P}\left(\;\frac{R^{\text{T}}w-E^{\text{T}}w}{\sqrt{w^{\text{T}}\Sigma w}}\geq \frac{-\gamma -E^{\text{T}}w}{\sqrt{w^{\text{T}}\Sigma w}}\;\right)=1-\text{F}\left(\;\frac{-\gamma -E^{\text{T}}w}{\sqrt{w^{\text{T}}\Sigma w}}\;\right)\;\geq \beta$$

or F $\;\left(\;\frac{-\gamma -E^{\text{T}}w}{\sqrt{w^{\text{T}}\Sigma w}}\;\right)\leq 1-\text{β}\;$, or. $\left(\;\frac{-\gamma -E^{\text{T}}w}{\sqrt{w^{\text{T}}\Sigma w}}\;\right)\leq F^{-1}\left(1-\beta \right)\;$

and finally

(12) $$E^{\text{T}}w+\;F^{-1}\left[\left(1-\text{β}\right)\right]\sqrt{w^{\text{T}}\Sigma w\;}\geq -\gamma$$

This analytic inequality is used in this research to formalize the probabilistic form of the risk for the optimization problems Eq. (5) and Eq. (8). It requires that the losses of the husbandry management expressed in return not to be bigger than value γ for the confidence interval *****β*****. Thus, if the decision-maker chooses a value for the probability *****β***** = 95%, the losses from the management will not be bigger than the predefined volume γ with a probability of 1–95% = 5% for their occurring.

## 5 Definition of portfolio problems from the economic results of the husbandry management

This research assesses several analytical models for the portfolio problems, which minimize the risk, applying the two formalization forms: standard deviation and losses. It has used real data from the National statistical institute of Bulgaria, related to the production and management of agriculture and animal husbandries. An excerpt from the statistic is given in Table 2 (Annual Report, 2020; Analysis, 2020).

**Table 2:** Excerpt of returns from agriculture and animal husbandries.

Return	2007	2010	2013	2017	Sum
Other grazing animals	4356.44	4158.42	3564.36	990.10	13069.32
Vegetables	10297.03	990.10	990.10	5148.51	17425.74
Viticulture	−1584.16	−30099.01	2970.30	9504.95	−19207.92
Dairy farming	6336.63	2574.26	3960.40	1386.14	14257.43
Orchard	1980.20	792.08	990.10	−1980.20	1782.18
Field crops	10693.07	12475.25	−16237.62	−24950.50	−18019.80
Pigs and porks	19801.98	7920.79	10891.09	39009.90	77623.76
Mixed	2574.26	2376.24	1584.16	2376.24	8910.90
Average per farm	6336.63	4158.41	-2178.22	-5544.55	2772.27
Total	60792.08	5346.53	6534.65	25940.59	98613.85

Using these historical data, the parameters for the portfolio problems, are evaluated according to relations Eq. (2) and Eq. (6). Below are defined five portfolio problems, which formalize the maximization of the return and minimization of risk. The risk is formalized in both forms as stochastic assessment of the volatilities and covariances, and the application of the probabilistic VaR approximation Eq. (12). The problem's solutions are giving the share of the resources, which are recommended for allocation per husbandry production. The received solutions are graphical interpreted, which is the reason the portfolio arguments to be decreased to two. But this is not a constraint for the general case of portfolio problem, which will evaluate the appropriate share of the resource, allocated per each husbandry product.

### 5.1 **Problem P1**: minimization of risk described as VaR approximation

The analytical definition of the portfolio problem with goal function for minimization of the risk, formalized as VaR relation Eq. (4), approximated analytically as inequality Eq. (12). This portfolio problem is the modification of Eq. (5) and takes the form

(13) $$\begin{array}{l} \begin{array}{l} \min\\ w \end{array}E^{\text{T}}w+\;F^{-1}\left[\left(1-\text{β}\right)\right]\sqrt{w^{\text{T}}\Sigma w\;}\\ w^{T}\left|1\right|=1,\;w^{T}\geq 0 \end{array}$$

This portfolio problem is well analytically defined. The parameters **E** and Σ are calculated in advance, according to relations Eq. (2) and Eq. (6) with the historical data of returns from the husbandry productions, illustrated in Table 3. For the case of graphical interpretation of the problem solutions, here the size of variables **w** are reduced to *N =* 2, which are components of local returns. The products chosen from Table 2 are “Milky cows” and “Pigs and pork”. The portfolio solutions define the allocation of resources, which should be invested for each product. The evaluated solutions respect the requirements for maximization of the return and minimization of the risk, formalized as VaR relation.

**Table 3:** Historical data of returns recorded for the husbandry components “milky cows” and “pigs and pork”.$$\Sigma =\left[\begin{array}{ll} \sigma_{11}^{2} & \sigma_{12}^{2}\\ \sigma_{21}^{2} & \sigma_{22}^{2} \end{array}\right]=\left[\begin{array}{ll} 0.0115 & -0.0282\\ -0.0282 & 0.4907 \end{array}\right]\;$$

Animal outcome component	2007	2010	2013	2017	Mean return *ME*_*i*_*i = 1,2*	Normalized *E*_*i*_*i = 1,2*	Standard deviation $\sigma_{i}$
**Milky cows**	6400	2600	4000	1400	3600	**0.16**	0.1074
**Pigs and porks**	19800	7900	10900	39000	19400	**0.84**	0.7005

The initial values of the portfolio problem about the mean return **E** and covariance matrix Σ are calculated according to relations Eq. (2) and Eq. (6) with the data from Table 3. These statistical parameters for the mean values of local returns *Ei,* standard deviations $\sigma_{i}$ , and the covariance matrix Σ are given in Table 3.

Because the returns *E*_*i*_ of these products are different in scale, they have been normalized by means to evaluate the portfolio solutions on a normalized scale. This allows the optimization problem Eq. (13) to give relative values of its solution **w.** Respectively, the problem solutions can be used without considering the real value of the resources, which will be allocated for the husbandry management per the different categories of the farm products. The normalization of the mean returns is evaluated according to the relation

(14) $$E_{i}=\;\frac{ME_{i}}{ME_{1}+\;ME_{2}}\;i=1,2.$$

where $ME_{i}$ are the mean values of returns from Table 3. Their normalized values are denoted in bold in Table 3 and relations Eq. (14) are applied for the analytical definition of the portfolio optimization problem.

The value of the inverse probability distribution function $F^{-1}\left[\left(1-\text{β}\right)\right]$ for Eq. (12) is taken from tables [URL3]. The probability level for the losses is assumed to be β=5% per year and the confidence level is (1−β)=95%. The value of the inverse is $F^{-1}\left[95\%\right]$= −1.645.

**Figure 4:**
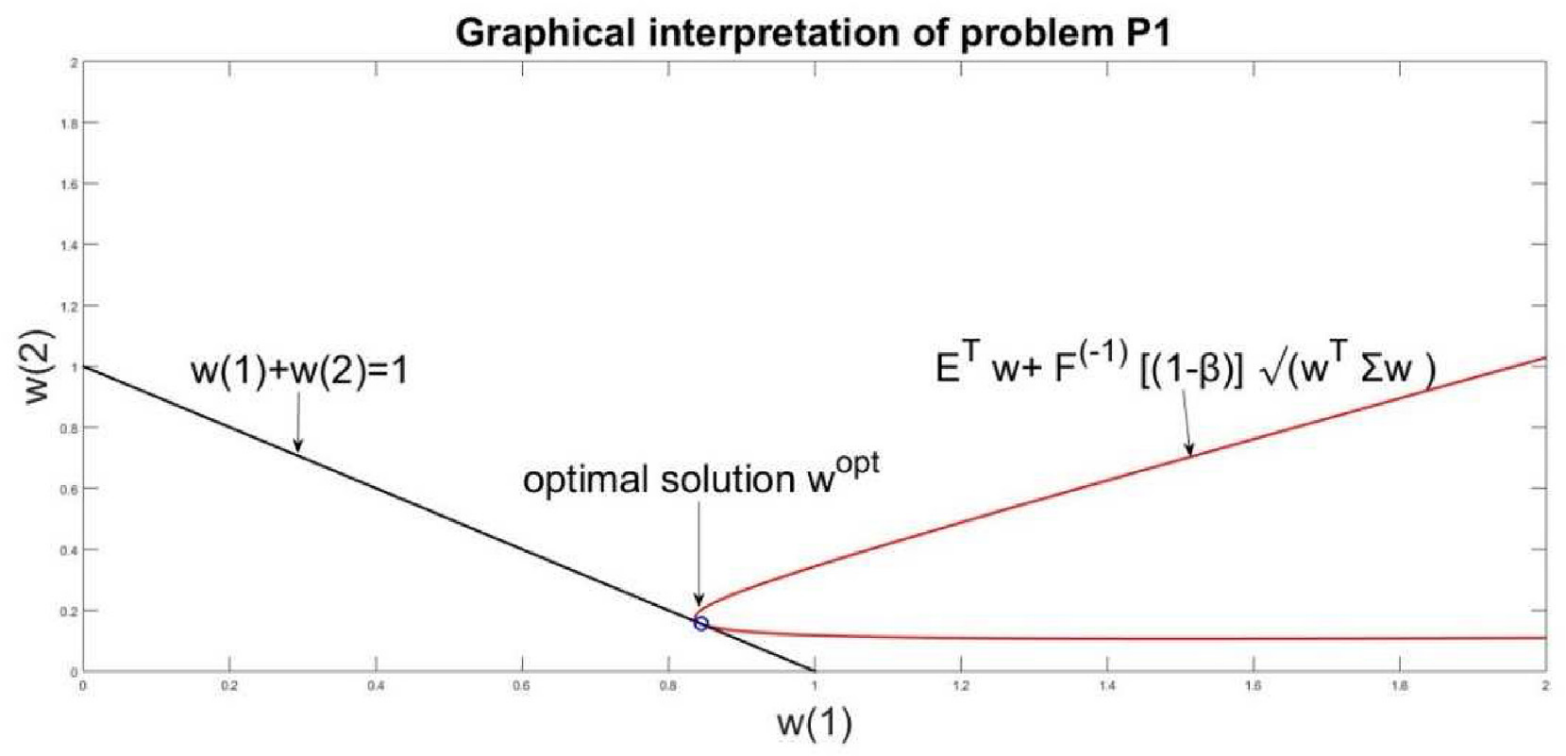
Graphical interpretation of problem's solution P1.

The solution of Eq. (13) recommends the decision-maker to allocate resources for these husbandry productions in relative shares as $w^{Topt}=\left(0.8447\;0.1553\right).$ Respectively the evaluated results for the husbandry management are for Risk=0.0126 ​andReturn=0.2632. The interpretation of these results says that the allocation of resources for husbandry productions has to be allocated to product “milky cows” equal to 84% and to products “pigs and pork” to 16%. This resource allocation will give a *Return* of 0.2632 and a *Risk* of 0.0126. For example, if we assume that the total resource contains the sum of mean returns: 3600 + 19400 = 23000, the recommendation is for “milky cows” to be allocated 0.84∗23000 = 19320 and for “pigs and pork” 0.16∗23000 = 3680. This allocation will increase the husbandry return by 26% and the risk of this allocation will be 1.26%. The value of the risk evaluated as a probabilistic VaR parameter is given by the value of the goal function of Eq. (13), evaluated at point $w^{Topt}$, which gives VaR = −0.0782. The negative value of VaR says that there will not occur losses for such an allocation of the investment resources. An additional criterion is the checks, applied by the relations, given in Figure 1. The real value of the husbandry return *R*(*t*) will be in the diapason [*Return* − *Risk, Return + Risk]* = [0.2506, 0.2758]. The lower bound E − σ = 0.2506 > 0 is a positive value, which proves that this allocation of the management resources will result in a positive return even in the worst case of the stochastic distribution of *R* (*t*). The graphical interpretation of problem solution is given in Figure 4.

The goal function is the minimal value of the algebraic relation ${\text{E}}^{\text{T}}\text{w}+{\text{ ​F}}^{-1}\left[\left(1-\right)\right]\sqrt{{\text{w}}^{\text{T}}\text{Σw ​}}$ equal to -0.0782. The optimal solution ${\text{w}}^{\text{opt ​}}$ has to lie on the line ${\text{w}}^{\text{T}}\left|1\right|=1$. The access point between these two lines is the solution ${\text{w}}^{\text{Topt}}=\left(0.8447\;0.1553\right)$, which is graphically presented in Figure 4.

### 5.2 **Problem P2**: minimization of the relation risk/return

The analytical definition of the optimization problem is in the form

(15) $$\begin{array}{l} \begin{array}{l} min\\ w \end{array}\;\frac{w^{T}\Sigma w}{E^{\text{T}}w}\\ w^{T}\left|1\right|=1,\;w^{T}\geq 0 \end{array}$$

This optimization problem has meaning to minimize the relation between the management risk $w^{T}\Sigma w$ and the management return $E^{\text{T}}w$**.** The difference with the problem Eq. (13) concerns the relation for the goal function. In Eq. (12) the goal function is in approximated form of the parameter Value at Risk, VaR. Problem Eq. (12) targets the minimization of risk without considering any level of return. In problem Eq. (15) the risk is assumed in its volatility form but the goal function takes into consideration simultaneously the minimization of risk and maximization of the management return. Problem Eq. (15) does not consider the VaR form of risk neither in the goal function nor in constraints.

**Figure 5:**
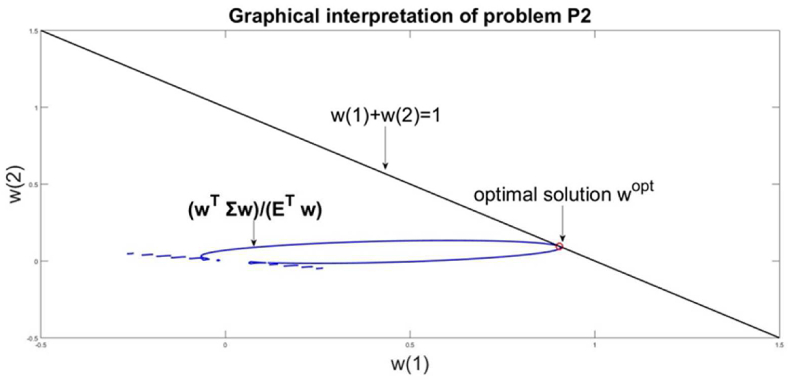
Graphical solution of problem P2.

The solution of this problem is $w^{Topt}=\left(0.9038\;0.0962\right),\;Risk=0.0090,\;Return=0.2226.\;$ The comparison with a problem Eq. (13) gives a decrease in the risk, but this results in decreasing the return too. The graphical interpretation of the problem solution is given in Figure 5. The access point between the line $w^{T}\left|1\right|=1\;$ and the nonlinear function $\frac{w^{T}\Sigma w}{E^{\text{T}}w}$ is the optimal solution to the problem.

### 5.3 Problem P3: minimization of the relation Risk/Return and considering VaR as an additional constraint

The analytical definition of the optimization problem is in the form

(16) $$\begin{array}{l} \begin{array}{l} min\\ w \end{array}\;\frac{w^{T}\Sigma w}{E^{\text{T}}w}\\ w^{T}\left|1\right|=1,\;w^{T}\geq 0-E^{\text{T}}w+\;1.645\sqrt{w^{\text{T}}\Sigma w\;}-\;\delta E^{\text{T}}w\;\leq 0 \end{array}$$

Relation Eq. (12) takes numerical value for the losses γ as part of the mean return of the husbandry management. The value of losses is defined as $\gamma =\delta E^{\text{T}}w$, which is a δ < 1 part of the total husbandry mean return $E^{\text{T}}w$**.** The value of the husbandry mean return is not known in advance, because the solutions **w** are not yet evaluated. That is why the relation in Eq. (16) takes a modified form in comparison with Eq. (12). For our numerical simulations, we choose the value of the coefficient δ=0.01 which means that the losses must be 1% of the total husbandry return. $E^{\text{T}}w.\;$

**Figure 6:**
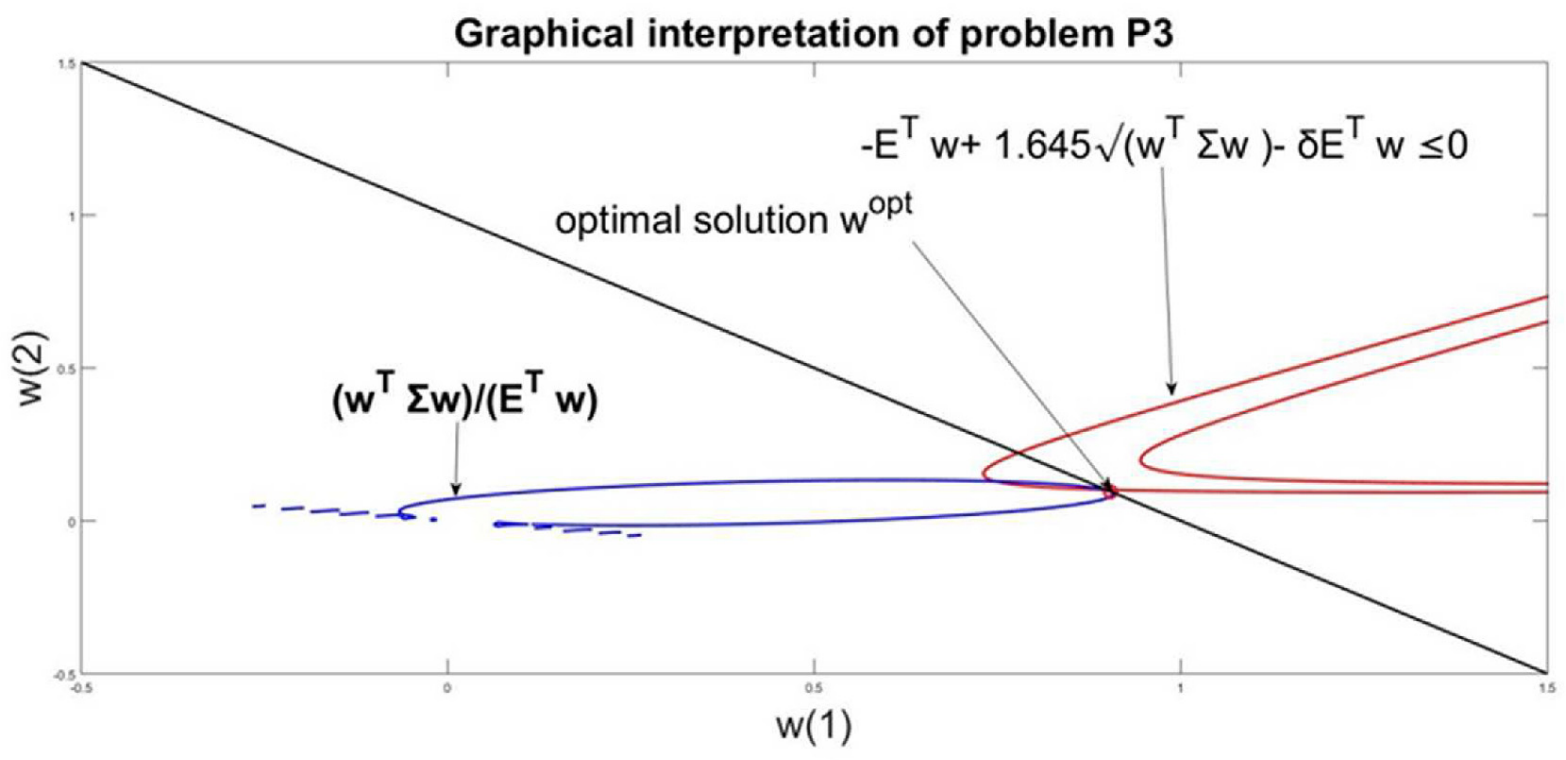
Graphical interpretation of the solution of problem P3.

The solution of problem Eq. (15) is $w^{Topt}=\left(0.9038\;0.0962\right),\;Risk=0.0090,\;Return=0.2226.\;$ The level of the loss is obtained to $\gamma =\delta E^{\text{T}}w=0.0022$. The graphical interpretation of the problem solution is given in Figure 6. The solution is the cross point of the constraints.

$$w^{T}\left|1\right|=1\;\text{and}-E^{\text{T}}w+1.645\sqrt{w^{\text{T}}\Sigma w\;}-\;\delta E^{\text{T}}w\leq 0$$

At this point, the graphics of the goal function $\frac{w^{T}\Sigma w}{E^{\text{T}}w}$ is a tangent one towards the line $w^{T}\left|1\right|=1$ and makes access to the point of the optimal solution.

It is seen that the relative amount of the management resources is recommended to be allocated to the component “milk and cows” which is pretty 90% of the resources. For the second component “pigs and pork” the recommendation is to allocate about 10% of the management resources. The risk and return for this optimization problem decrease in comparison with the problem Eq. (13).

The graphical interpretation of problem solutions shows that the relation $-E^{\text{T}}w+\;1.645\sqrt{w^{\text{T}}\Sigma w\;}$ − $\;\delta E^{\text{T}}w\;\leq 0$ crosses the linear relation $w^{T}\left|1\right|=1$ in two points. These points define the feasible domain for the optimization problem Eq. (16). The optimal solution is defined by the goal function, which has to access one of the feasible points. For problem Eq. (16) the goal function has the same form as in problem Eq. (15). Currently, the goal function defines the same solution as Eq. (15). Thus, the VaR constraints currently do not influence the solution of Eq. (16) and it is equal to this one of Eq. (15).

### 5.4 **Problem P4**: *the modified problem for minimizing the risk as a volatility*

This problem evaluates the resource allocation **w**, which targets simultaneously minimization of the management risk $w^{\text{T}}\Sigma w$ and maximizes the return $E^{\text{T}}w$. This problem doesn't contain the VaR relation neither in the goal function nor as constraints. The parameter λ gives the relative weights of the risk and return in the optimization problem Eq. (17). The values of λ are normalized in the domain [0, 1]. Each value of λ results in a different solution **w** (λ) and respectively with different values of *Risk(*λ) and *Return(*λ)*.*

(17) $$\begin{array}{l} \begin{array}{l} min\\ w \end{array}\left\{\left(1-\lambda \right)w^{\text{T}}\Sigma w-\text{λ}E^{\text{T}}w\right\}\\ w^{T}\left|1\right|=1,\;w^{T}\geq 0 \end{array}$$

The parameter λ expresses the ability of the manager to undertake risk in management decisions. The parameter λ concerns the individual, subjective assumptions of the decision-makers and it does not exist a unique optimal value for it. A practical approach is to solve a set of problems Eq. (17) for different λ and from the obtained solutions to choose one, which is accepted as appropriate for the decision-makers. Another approach, practically applied in [Khan et al., 2020] is to choose this solution $w\left(\lambda^{*}\right)$, which gives the minimum value of the Sharpe relation, *Risk(*$w(\lambda^{*})/Return(w(\lambda^{*})=w^{\text{T}}(\lambda^{*})\Sigma w(\lambda^{*}/E^{\text{T}}w(\lambda^{*})$, where $w\left(\lambda^{*}\right)\;$ is the solution of Eq. (17) for the value $\lambda^{*}$, giving minimal Sharpe coefficient.

For this research we have been solved 101 problems Eq. (17) changing λ from 0 to 1 with step 0.01, λ = [0; 0.01; 1]. The resulting solution of Eq. (17) is: $\lambda^{*}=0.02;\;w^{\text{T}}\left(\lambda^{*}\right)=\left(0.9037\;0.0963\right)$; *Risk (*$w(\lambda^{*})=0.0091;\;Return\left(w(\lambda^{*}\right)=0.2227$; *Risk(*$w(\lambda^{*})/Return(w(\lambda^{*})=0.0409$*.*

**Figure 7:**
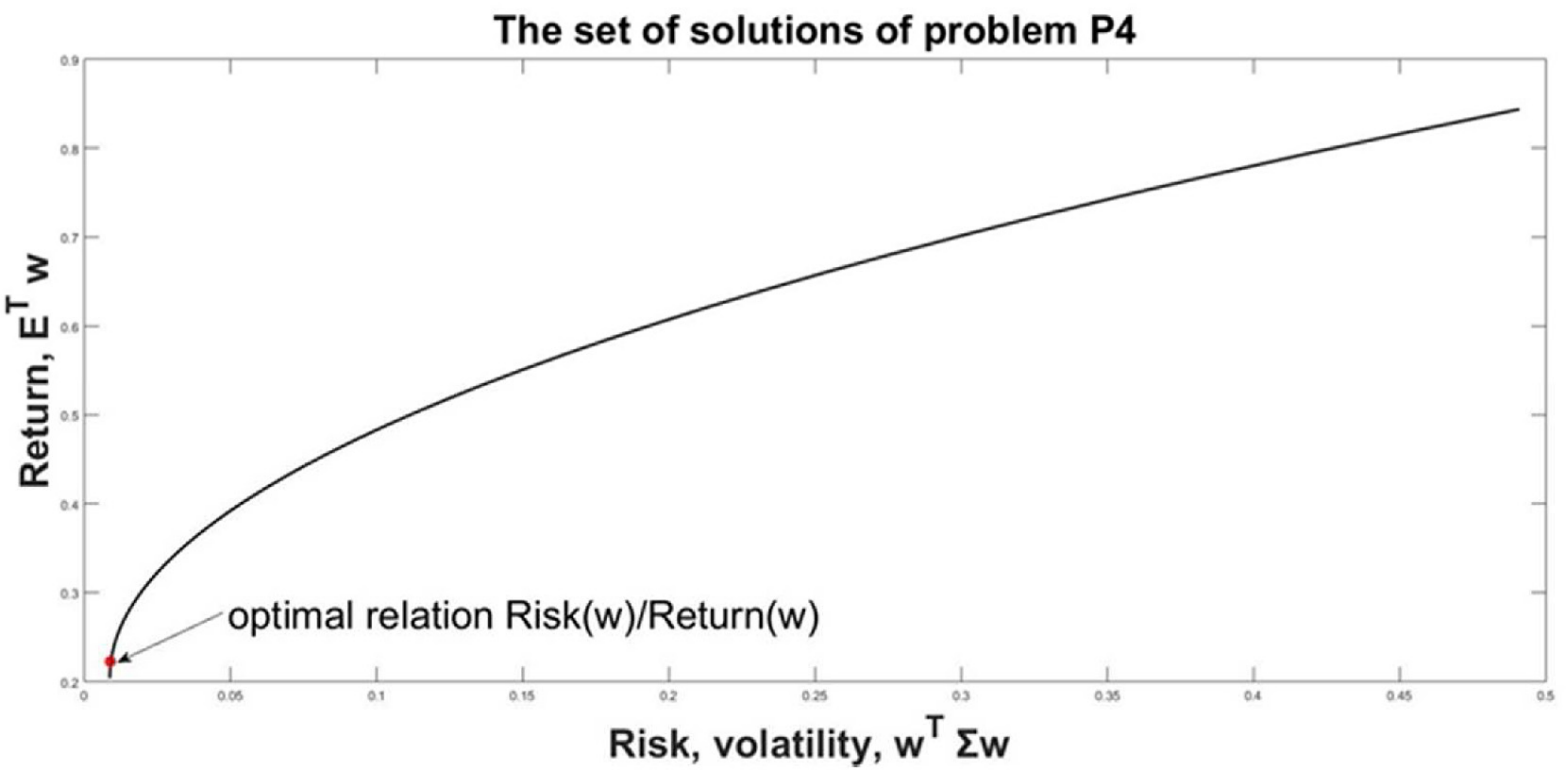
The set of solutions for problem P4 and the chosen solution for $w\left(\lambda^{*}\right)$.

**Figure 8:**
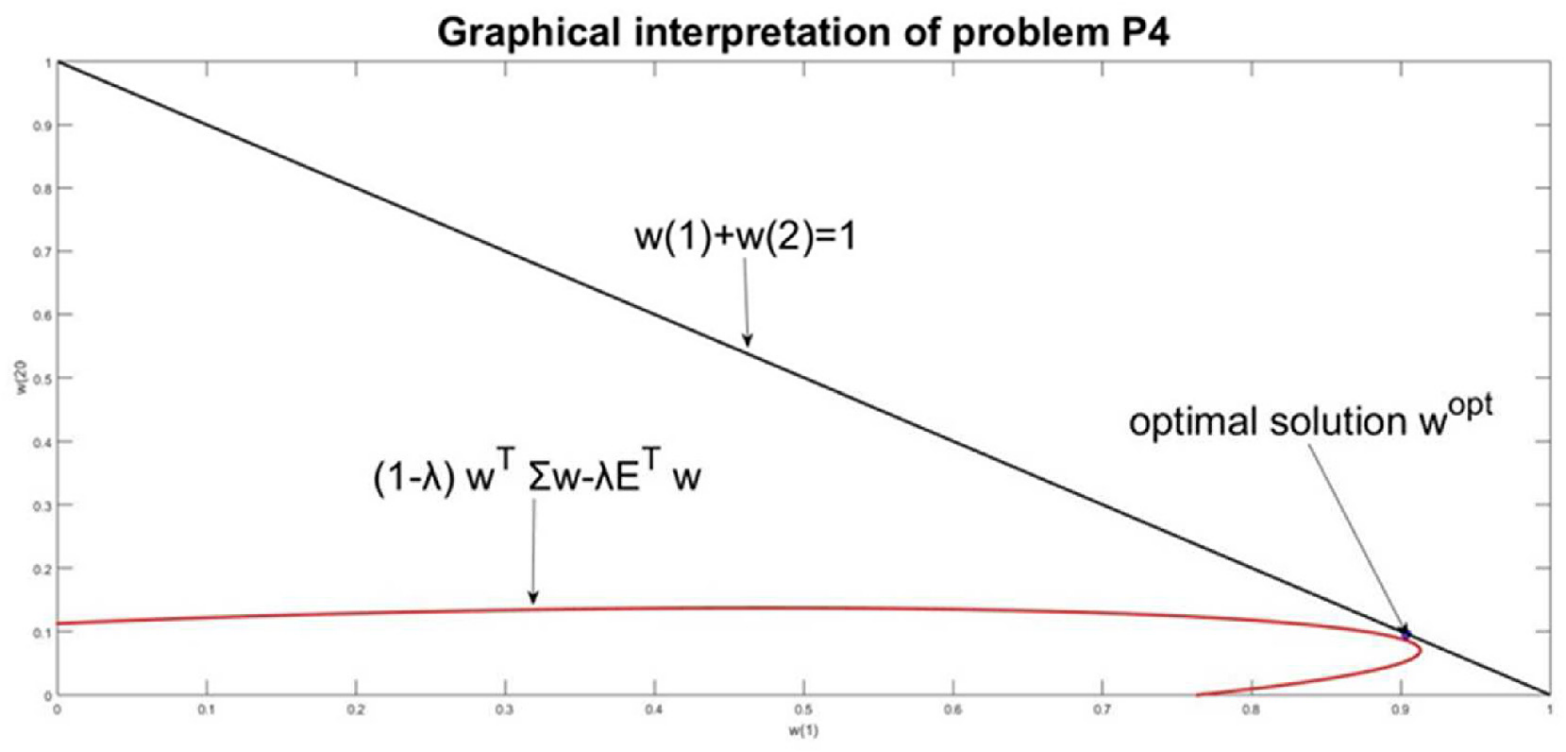
Graphical interpretation of the solution of problem (17).

In Figure 7, the set of solutions of Eq. (17) is presented as an “efficient frontier” in the plane *Return (Risk)* and the resulting point with the $w\left(\lambda^{*}\right)$. The graphical interpretations of the goal function and constraints of Eq. (17) are given in Figure 8. The solution to this problem will be compared with the ones of the previously defined problems.

### 5.5 Problem P5: the modified problem for minimization of the risk as volatility with nonlinear VaR constraint

This problem is defined with the goal function of Eq. (17) but the set of constraints includes additionally the VaR approximation Eq. (12). The same value of δ=0.01 as in problem Eq. (16) is applied, which means that the value of the loss is established at 1% of the total husbandry return $E^{\text{T}}w.$

(18) $$\begin{array}{l} \min_{w}\left\{\left(1-\lambda \right)w^{\text{T}}\Sigma w-\text{λ}E^{\text{T}}w\right\}\\ w^{T}\left|1\right|=1,\;w^{T}\geq 0-E^{\text{T}}w+\;1.645\sqrt{w^{\text{T}}\Sigma w\;}-\;\delta E^{\text{T}}w\;\leq 0 \end{array}$$

**Figure 9:**
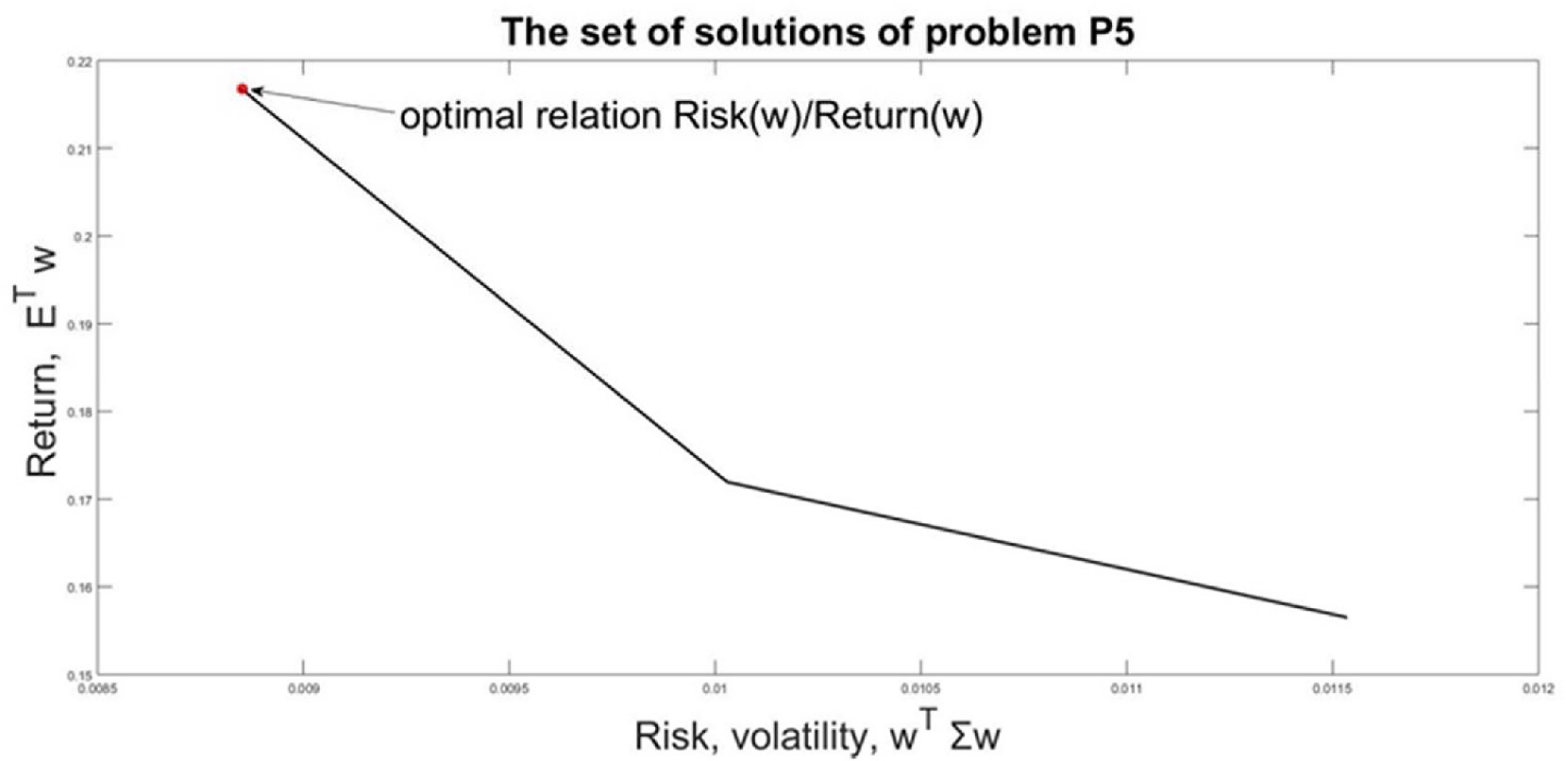
The set of solutions of P5 and the chosen solution for $w\left(\lambda^{*}\right)$.

The parameter λ gives again different relative weights to the risk and return in the optimization problem Eq. (18). Problem Eq. (18) is solved with a set of λ[0,1]. The difference between the current problem Eq. (18) and the previous one Eq. (17) is that Eq. (18) does not have a solution for each value of λ. The set of solutions of Eq. (18) is narrow, due to the restrictions, generated by the VaR nonlinear constraint. The feasible solutions and the optimal one, which has the maximal value of the relation *Risk(*λ)/*Return(*λ) are given in Figure 9.

The solutions of Eq. (18) are: $\lambda^{*}=0.49;\;w^{\text{T}}\left(\lambda^{*}\right)=\left(0.91240.0876\right)$**;** *Risk(*$w(\lambda^{*})=0.0089;\;Return\left(w(\lambda^{*}\right)=0.2167$; *Risk(*$w(\lambda^{*})/Return(w(\lambda^{*})=0.0411$*.*

**Figure 10:**
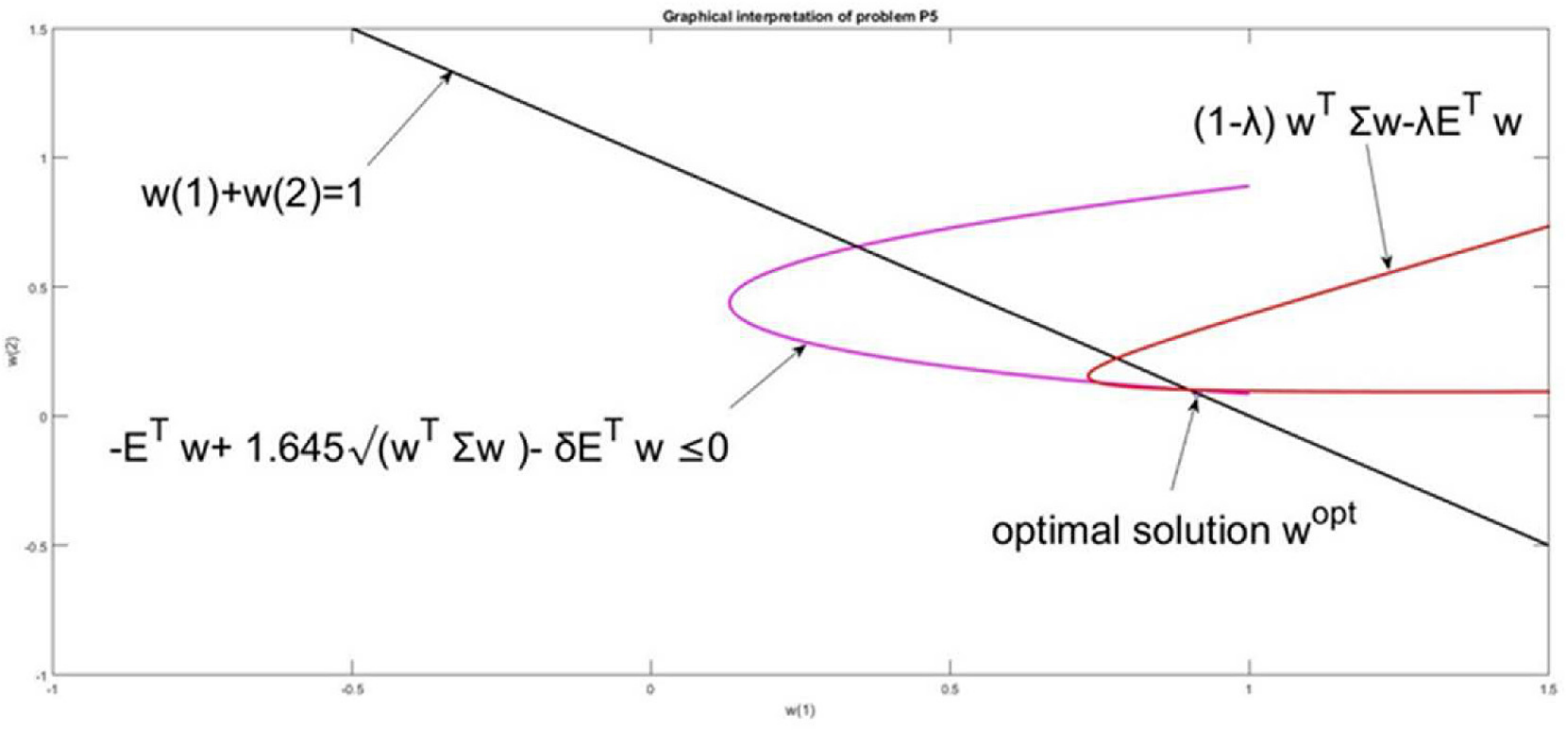
Graphical interpretation of the solution of problem (18).

The graphical interpretation of the goal function and constraints of problem Eq. (18) are presented in Figure 10.

The problem solution is the cross point between the constraint $w^{T}\left|1\right|=1$ and the VaR approximation inequality Eq. (12). Two intersection points exist for the simultaneous requirements of these constraints. The goal function defines the unique point, which is the solution to the optimization problem.

The solutions to these five defined problems for resource allocation by risk minimization and maximization of return by the management of husbandry are summarized in Table 4.

**Table 4:** Solutions of problems **P1** to **P5**.

Problems	P1	P2	P3	P4	P5
**W**	0.8447	0.9038	0.9038	0.9037	0.9124
	0.1553	0.0962	0.0962	0.0963	0.0876
**Risk**	0.0126	0.0090	0.0090	0.0091	0.0089
**Return**	0.2632	0.2226	0.2226	0.2227	0.2167
**Risk/Return**	0.0479	0,0404	0.0404	0.0409	0.0411

Analysis of these results is given in the next section.

## 6 Discussions

**Figure 11:**
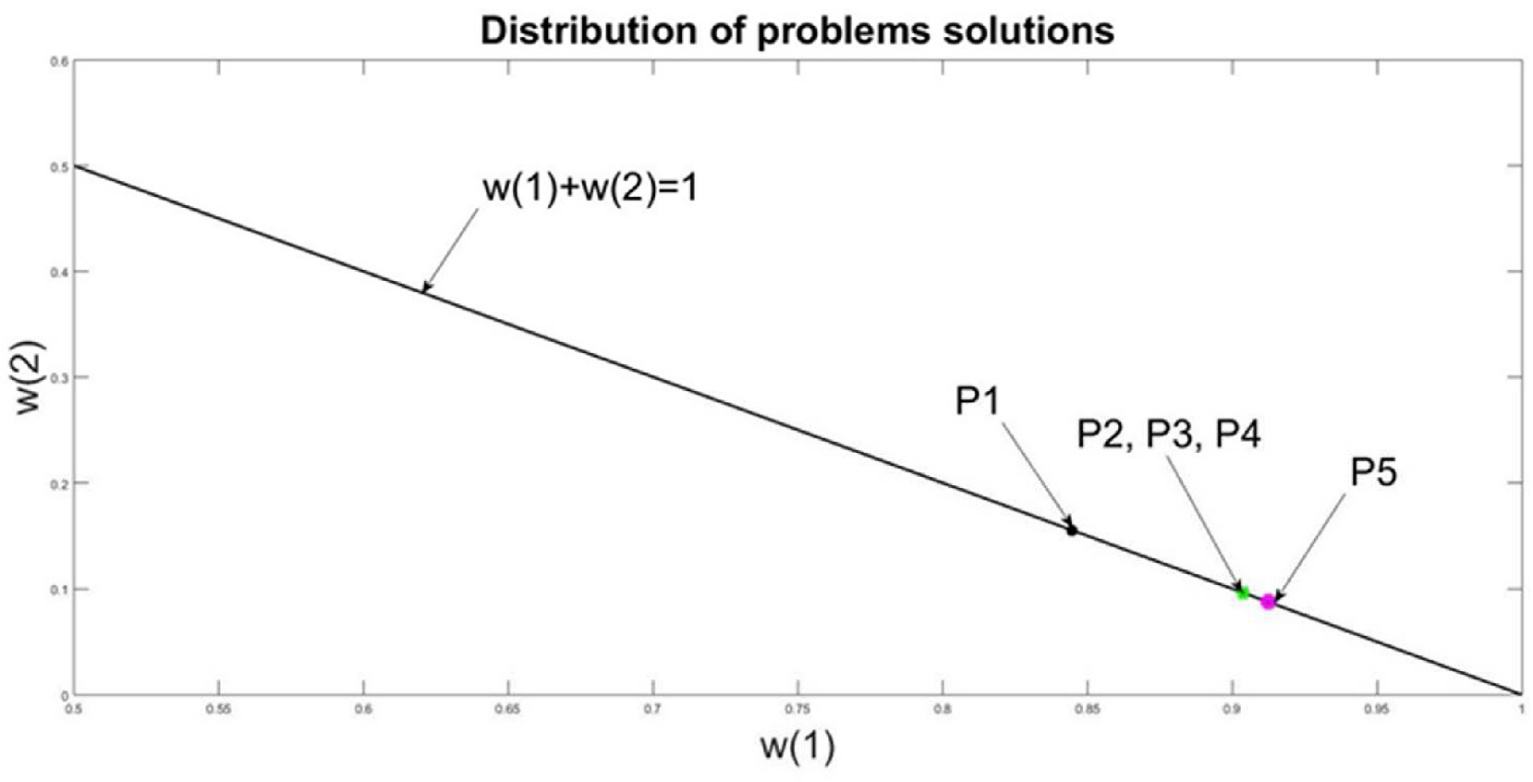
Distribution of problems solutions.

These results illustrate that the inclusion of VaR constraint in a portfolio problem decreases additionally the risk, in comparison with the problems, which formalize the risk only in the statistical form of the volatility. The decrease of the risk leads to a decrease in the return, which is common relation in the portfolio theory. Problems P2 and P4 are defined in classical portfolio form and their solutions are used as benchmarks for comparisons of the new modified portfolio problems P1, P3, and P5. To provide a common comparison between the solutions of these five portfolios models the Sharpe ratio is evaluated as a relation between risk and return. The minimal Sharpe ratio keeps a minimal value of risk and maximal return. The minimal Sharpe value is obtained for problems P2 and P3, which have very close solutions. But problem P5 achieves the lowest value of risk because it applies simultaneously both formalization of risk as volatility and loss. This is an additional benefit for the decision maker. According to the relations between risk and return it is natural for a lower risk to obtain a lower return, which is evident from the comparison of problems P3 and P5. The graphical interpretations of the problem solutions are presented in Figure 11 in the space of w (1) and w (2). The solutions of problems P2 and P5 are very close to these of the classical portfolio problem P3. The values of their risks are low due to the explicit inclusion of the VaR constraint Eq. (12) in their feasible domains. But the lower level of risk corresponds to a low level of return in comparison with the other problems P1 and P4.

We can see a close relationship between problems P2 and the modified P3. Both problems have the same goal functions, defined by the relation *Risk(***w***)/Return(***w***)*

$$\begin{array}{l} min\\ w \end{array}\;\frac{w^{T}\Sigma w}{E^{\text{T}}w}.$$

This relation strongly influences problem solutions, which are equal for P2 and P3 despite that in P3 the VaR constraint Eq. (12) is included explicitly as a constraint. Problem P1 has the highest value of risk. This is related to the problem's definition of P1. The goal function targets only risk minimization, which is formalized as VaR approximation in form Eq. (12). This problem does not consider the risk as volatility of the returns. In Figure 11 the solutions to the five problems are presented in the space w (2)/(w (1))and all of them are lying on the line $w^{T}\left|1\right|=1$.

The tendency of problem solutions is to allocate a bigger value to w (1) for the component “milk cows”. The allocation of resources to component w (2) is about 10% of the husbandry return. The comparison of the problem solutions can be assessed also by the relation *Risk(***w***)/Return(***w***),* which gives an advantage to the modified problem P3.

Such comparison recommends the resource allocations to be performed, according to the solution of problem P3 because the result in a risk of 0.0404 is the lowest one and the Sharpe ratio is minimal. But the risk can be reduced additionally by definition of problem P5 with simultaneous usage of risk formalization as a standard deviation of return and Loss.

## 7 Conclusions

The research presents quantitative models for resource allocation in animal husbandry management. The resources, which are allocated per different production, are evaluated, applying the formal modeling of portfolio theory. The defined optimization problems considered minimization of the risk and maximization of the return from the husbandry productions. Five portfolio problems are defined. This research did modifications of the portfolio problems. It has added additional constraints, which formalize the risk with a value of a loss, which can occur in probability. The formal definition of this constraint comes from the definition of the parameter VaR. The probability formalization of VaR is approximated with algebraic relation, which was used as a goal function and constraint for the modified portfolio problems. The modified portfolio problems target maximization of return and minimization of risk, formalized by two parameters: volatilities and loss. Such additional consideration of risk allows reducing additionally the risk in resource allocation for the husbandry productions. The research graphically illustrates the potential benefits from the simultaneous usage in two forms the risk formalization in a portfolio problem. The graphical approach is made only with two products but the portfolio problem can accommodate any number of productions of husbandry.

The modifications of the portfolio problems can be additionally complicated with constraints, which concern resource allocation for tasks such as inventory management, insurance policies, and infrastructure disbursements. Because the resource allocation is performed in the framework of the portfolio theory the expected results can decrease the risk in management decisions and provide a sustainable increase in the returns.

A potential complication in the formal modification of the portfolio problems could be its definition as a bi-level optimization problem. The bi-level optimization problems give advantages in considering simultaneously two goal functions, and an extended set of constraints. The bi-level solutions contain an extended set of arguments, which give optimal values for more management parameters and this can benefit the decision-making process in resource allocation.

## Declarations

### Author contribution statement

Todor Stoilov: Conceived and designed the experiments; Performed the experiments; Wrote the paper.

Krasimira Stoilova: Performed the experiments; Analyzed and interpreted the data; Wrote the paper.

Stanislav Dimitrov: Contributed reagents, materials, analysis tools or data.

### Funding statement

Prof Todor Stoilov was supported by Ministry of Education and Science-Bulgaria [N Д01-62/18.03.2021].

### Data availability statement

Data associated with this study has been deposited at Analysis, 2020 Analysis of agriculture development and food industry in Bulgaria, SWOT analysis, Agricultural Academy, Bulgaria. Institute of Agrarian Economics. 2020 (in Bulgarian). https://www.mzh.government.bg/media/filer_public/2020/01/21/analiz_na_sstoianieto_na_selskoto_stopanstvo_i_khranitelno-vkusovata_promishlenost_izgotven_ot_institut_po_agrarna_ikonomika.pdf

Annual Report, 2020 Annual report about the agriculture developments in Bulgaria, Ministry of Agri-culture, 2020 (in Bulgarian). https://www.mzh.government.bg/media/filer_public/2020/12/03/agd_2020_web.pdf

### Competing interest statement

The authors declare no conflict of interest.

### Additional information

No supplementary file.
